# Obstructive Sleep Apnea in Adults: What Primary Care Physicians Need to Know

**DOI:** 10.7759/cureus.17843

**Published:** 2021-09-09

**Authors:** Enrique Arredondo, George Udeani, Ladan Panahi, Pahnwat T Taweesedt, Salim Surani

**Affiliations:** 1 Pharmacy, Irma Lerma Rangel College of Pharmacy, Texas A&M University, Kingsville, USA; 2 Pulmonary Medicine, Corpus Christi Medical Center, Corpus Christi, USA; 3 Anesthesiology, Mayo Clinic, Rochester, USA; 4 Medicine, Texas A&M University, College Station, USA; 5 Medicine, University of North Texas, Dallas, USA; 6 Internal Medicine, Pulmonary Associates, Corpus Christi, USA; 7 Clinical Medicine, University of Houston, Houston, USA

**Keywords:** nonsurgical treatment, obstructive sleep apnea (osa), risk factors for obstructive sleep apnea (osa), osa, continous positive airway pressure, bilevel positive airway pressure, pathophysiology, comorbidities, surgical treatment

## Abstract

Obstructive sleep apnea (OSA) remains a prominent disease state characterized as the recurrent collapse of the upper airway while sleeping and is estimated to plague 936 million adults globally. Although the initial clinical presentation of OSA appears harmless, it increases the risk of cardiovascular diseases such as heart failure, stroke, and hypertension; metabolic disorders; and an overall decrease in quality of life, in addition to increasing mortality. Current treatment of OSA includes lifestyle changes, behavioral modification, mandibular advancement devices, surgical treatment, and continuous positive airway pressure, which remains the gold standard. It is crucial to identify OSA early on and initiate treatment to mitigate the adverse health risks it imposes. This review will discuss the pathophysiology, epidemiology, management strategies, and medical treatment of OSA.

## Introduction and background

**Figure 1:**
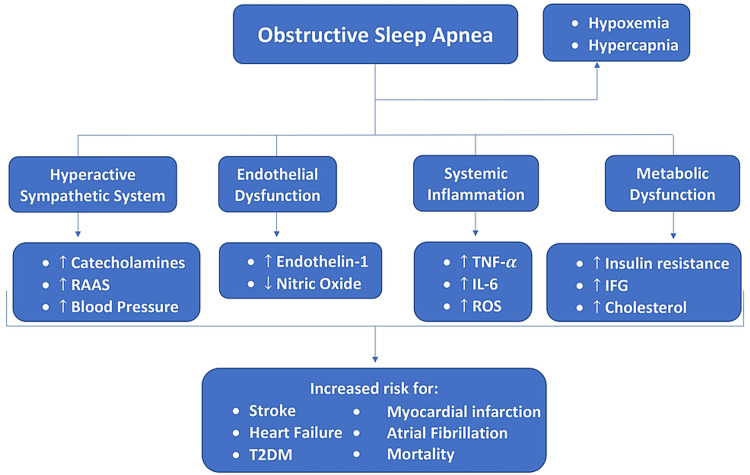
The multifactorial pathogenesis of OSA and how the combined effects can increase the risk of outcomes. ↑ = increased; ↓ = decreased; RAAS = renin-angiotensin-aldosterone system; TNF-α = tumor necrosis factor alpha; IL-6 = interleukin 6; ROS = reactive oxygen species; IFG = impaired fasting glycemia; T2DM = type 2 diabetes mellitus; OSA = obstructive sleep apnea

Obstructive sleep apnea (OSA) refers to recurrent upper airway narrowing or collapsing during sleep, causing cyclic episodes of hypoxemia, hypercapnia, and awakenings [1]. The clinical presentation of OSA usually involves snoring, daytime somnolence, narcolepsy, and cognitive impairment. Because the clinical presentation of OSA does not include alarming symptoms, it can remain undiagnosed. If left untreated, it can significantly increase the risk of cardiovascular diseases (CVD), including hypertension, atrial fibrillation, heart failure, stroke, pulmonary hypertension, and myocardial infarction [2-6]. It is estimated that OSA affects 40-60% of patients with CVD [7]. In addition to the cardiovascular risks, type 2 diabetes, hyperlipidemia, and hypertriglyceridemia are commonly seen in this patient population [8]. Some proposed pathophysiology mechanisms that contribute to the overall risk include hyperstimulation of the sympathetic nervous system, endothelial dysfunction, systemic inflammation, and metabolic dysfunction [2,8]. Figure 1 illustrates the correlation as to how the multifactorial risks contribute to possible outcomes.

## Review

Pathophysiology

Sympathetic Nervous System

In a normal physiological process, the parasympathetic system is dominant during sleep. However, due to increased carbon dioxide and decreased oxygen from the frequent collapse of the upper airway, peripheral and central chemoreceptor activation leads to increased sympathetic output [8,9]. This increase in sympathetic output persists even when awake. Narkiewicz et al. compared the sympathetic output of obese individuals with and without OSA. They reported that obesity alone, in the absence of OSA, was not accompanied by increased sympathetic output suggesting that OSA is an independent risk factor for increased catecholamine activity [10]. Due to the innervation of the renal system by the sympathetic system, the renin-angiotensin-aldosterone system (RAAS) becomes overstimulated as well. OSA patients tend to have higher angiotensin II and aldosterone levels leading to water retention and peripheral vasoconstriction [11]. Consequently, many individuals with OSA have high blood pressure [12].

Endothelial Dysfunction

Endothelium cells play a role in the regulation of vascular tone by releasing vasoactive and vasorelaxant factors. However, in the state of OSA, there appears to be a disruption in the function of the endothelium [13]. Phillips et al. conducted a prospective study among OSA patients by measuring hemodynamics, oxygen saturation, and circulating endothelin-1. They found that OSA patients have increased blood pressure and endothelin-1 levels, both of which were reduced after treatment [14,15]. Nitric oxide, a vasodilator, also appears to be affected in the state of OSA. Although circulating serum nitric oxide is suppressed, it is reversible on initiating nasal continuous positive airway pressure (CPAP) [16].

Systemic Inflammation

OSA can be viewed as a low-grade chronic inflammatory disease caused by an increase in circulatory serum inflammatory markers such as tumor necrosis factor-alpha (TNF-a) and interleukin-6 (IL-6) [17-19]. In addition, the hypoxia produced by the intermittent collapse of the upper airway leads to increased production of reactive oxygen species (ROS) [20]. The combination of increased pro-inflammatory cytokines and ROS may contribute to the increased risk of CVD and overall mortality.

Metabolic Dysfunction

Along with the increased inflammatory state caused by OSA, most patients display a higher prevalence of type 2 diabetes. The Sleep Heart Health Study, a cross-sectional analysis of 2,588 subjects, demonstrated an association between OSA and impaired fasting glucose, impaired glucose tolerance, and occult diabetes [21]. Furthermore, a meta-regression analysis conducted by Nadeem et al. found that OSA patients have increased total cholesterol, low-density lipoprotein, and triglycerides [22]. Increased blood glucose and dyslipidemia are both risk factors for CVD.

Epidemiology

OSA remains a prevalent global disease prominently seen in the middle-aged and elderly population. Benjafield et al. conducted a literature-based analysis involving 16 countries utilizing the American Academy of Sleep Medicine 2012 diagnostic criteria. They estimated that 936 million adults (both men and women) aged 30-69 years have mild-to-severe OSA, and 425 million adults aged 30-69 years have moderate-to-severe OSA globally [23]. Despite recent analysis estimating nearly 1 billion adults with OSA globally, it remains highly underrecognized, leading to a vast proportion of adults being undiagnosed. Another study highlighted that in the United States, 82% of men and 93% of women are living with OSA undiagnosed [24,25]. Due to the multifactorial pathogenesis of OSA, it creates a heavy social and economic burden. In 2015, the estimated cost for undiagnosed OSA in the United States was $149.5 billion, with $30 billion attributed to the increased risk of comorbidities (e.g., hypertension, CVD, and diabetes) and $86.9 billion to lost productivity. During the same year, it was estimated that the cost for diagnosing and treating OSA in the United States was $12.4 billion [26,27].

Risk factors for OSA include obesity, gender, age, medications, and alcohol use (Table 1) [24]. It is essential to understand the epidemiology of OSA to efficiently screen high-risk patients and initiate effective treatment to prevent the increased health risks and economic burden.

**Table 1:** Summary of the proposed pathophysiological mechanisms for the risk factors.

Risk factor	Proposed pathophysiological mechanism
Obesity	Adipose deposition at the upper airway leads to an increased risk of collapsibility and decreased patency
Gender	Males have increased adipose tissue deposition at the neck and longer airways, which make the airway collapse easily
Age	Aging leads to decreased genioglossus response to negative pressure and increased type I collagen, which delay the contractile-relaxant response
Medications and alcohol use	Medications such as opioids, benzodiazepine, baclofen, and alcohol can cause upper airway muscle relaxation leading to upper airway collapse

Obesity

Historically, obesity has been identified as the critical risk factor for the development and progression of OSA [28]. Obesity has a direct correlation to OSA because of the deposition of adipose around the upper airway and neck, which increases the risk of pharyngeal collapse, altering both mechanical and neural control and leading to OSA [29]. Peppard et al. demonstrated that a 10% increase in body weight can lead to a six-fold increase in moderate-to-severe OSA and the Apnea-Hypopnea Index (AHI) [24,30]. The Harvard T.H. Chan School of Public Health has predicted that by 2030 about one in two adults in the United States will be obese (48.9%), with nearly 25% having severe obesity [31]. As the obesity epidemic starts to worsen, the prevalence of OSA is also expected to rise [32].

Gender

Males are more predisposed to OSA compared to females. This observation was demonstrated in the Study of Health in Pomerania (SHIP)-Trend study with 1,280 subjects. The prevalence of OSA was 59% in males and 33% in females [33]. Whittle et al. demonstrated that males tend to have higher neck fat deposition than females utilizing magnetic resonance imaging [34]. Due to the increased neck circumference from fat deposition, males tend to have a higher risk for upper airway collapse compared to females. In addition, males have longer airways compared to females. It has been hypothesized that due to the more extended airway anatomy, males are more predisposed to pharyngeal collapse [35]. In general, females have been shown to have less severe OSA incidence compared with males having similar body mass index (BMI) [36].

Age

Age is a risk factor for OSA. It is predominantly seen in middle-aged and elderly individuals (both men and women). The SHIP-Trend study demonstrated that the prevalence of AHI increased at a continuous rate with age in both men and women, and AHI severity did not increase until after the age of 50 [33]. The proposed pathophysiological mechanisms for OSA prevalence increasing with age include the decrease in genioglossus response to negative pressure, which prevents dilator muscles to compensate from a collapsing perturbation, and increased type I collagen in the pharyngeal constrictor muscle, which may be the cause for delayed contractile-relaxant response [37,38]. According to the 2019 World Population Ageing by the United Nations: Department of Economic and Social Affairs, by 2050, the ratio of people over 65 years old globally is expected to increase to one in six compared to one in eleven in 2019 [39]. Given the rise in both age and obesity in the future, OSA prevalence is expected to increase.

Medications and Alcohol Use

Certain medications have been shown to increase the risk of OSA. Medication use during anesthesia such as hypnotics, analgesics, and sedatives can increase the risk of OSA and perioperative respiratory compromise. Benzodiazepines can result in airway narrowing due to relaxation and decrease central respiratory activity [24]. Opioids can also decrease respiratory rate, depth of respiration, and respiratory drive, and increase chest wall rigidity. Baclofen can cause muscle relaxation leading to upper airway narrowing. Alcohol consumption is one of the significant modifiable risk factors of OSA, although results are conflicting. Alcohol consumption is thought to cause genioglossal muscle tone reduction leading to upper airway collapse.

Signs and symptoms of obstructive sleep apnea

The history from the patient’s spouse or family members is very important. The patient or spouse may report the patient snoring and observed apnea. The patient may complain of feeling tired, sleepy, exhausted, and run-down. In addition, the patient may complain of morning headaches, dry mouth, sore throat, and sleep fragmentation. On examination, the patient may have a high BMI, retrognathia, crowded pharyngeal space, and a high Mallampati score (Table 2) [1,7,24]. Screening can be accomplished by the use of the snoring, tiredness, observed apnea, high BP, BMI, age, neck circumference, and male gender (STOP-BANG) or the Berlin questionnaires [1,8,24]. The STOP-BANG questionnaire comprises yes/no responses to questions such as snoring loudly, feeling tired/sleepy, observed apnea, having high blood pressure, high BMI >35 kg/m^2^, age >50 years, neck circumference >40 cm, and male gender. Positive screening suggests further diagnostic testing with overnight polysomnography, which can be a home study or an in-laboratory study [24].

**Table 2:** Signs and symptoms of obstructive sleep apnea.

Signs and symptoms of obstructive sleep apnea
Fatigue
Tiredness
Excessive daytime sleepiness
Loud snoring
Observed apneas
Sleep fragmentation
Early morning headache
Dry mouth and sore throat
Drowsiness
Crowded pharyngeal space

Management and treatment of obstructive sleep apnea

Behavioral and Lifestyle Changes

Because obesity and neck fat deposition are directly correlated to the development and progression of OSA, weight loss is the primary target. The AASM recommends targeting a BMI of 25 kg/m^2^ or less, with weight loss recommended to all overweight patients [40]. In 2019, a randomized clinical trial studied the effectiveness of an intensive weight loss program for patients with severe OSA. The study concluded that through an intensive weight loss program, patients with obesity and severe OSA benefited from reduced weight and OSA severity. Secondary outcomes included improvements in lipid profiles, glycemic control, and inflammatory markers [41]. Another adjunct therapy to OSA is positional therapy. For patients with OSA, sleeping in a supine position may increase the probability of airway collapse due to unfavorable airway geometry, reduced lung volume, and the inability of airway dilator muscles to compensate for the collapse [42]. The AASM recommends using a positioning device such as a pillow, tennis ball, or backpack to help keep the patient in a non-supine position [40].

Continuous Positive Airway Pressure

CPAP prevents upper airway collapse by acting as a pneumatic splint through constant positive pressure from a mask interface [43]. CPAP continues to be the gold standard treatment for OSA due to its ability to improve the quality of life, improve daytime somnolence, and lower blood pressure [44-46]. The AASM currently recommends CPAP for moderate-to-severe and mild OSA patients [40]. However, although CPAP has been demonstrated to improve blood pressure, it has not been shown to improve cardiovascular outcomes. The Sleep Apnea Cardiovascular Endpoints (SAVE) randomized clinical trial evaluated CPAP in OSA patients for the secondary prevention of cardiovascular outcomes. The trial included 2,717 moderate-to-severe OSA patients with a mean follow-up of 3.7 years. CPAP significantly improved daytime sleepiness and decreased hypopnea events. When assessing cardiovascular outcomes, there was no difference between the control and treatment groups [47]. In patients with a history of OSA, CPAP or noninvasive positive pressure ventilation is recommended to be continuously used during preoperative treatment, which may reduce postoperative cardiovascular complications.

Mandibular Advancement Devices

Although CPAP remains the gold standard, an alternative treatment for individuals who cannot tolerate CPAP treatment is mandibular advancement devices (MADs). MADs are oral appliances that shift the position of the mandible forward and alter the position of both the tongue and jaw, further preventing the collapse of the upper airway [48]. A systematic review and meta-analysis study compared CPAP to MAD in blood pressure outcomes. It was found that in patients with OSA, the use of CPAP or MAD was associated with reductions in blood pressure. In addition, there was no statistical difference between blood pressure outcomes between the two treatments [49]. The Academy of Dental Sleep Medicine and AASM released practice guidelines in 2015 on using MADs in OSA. It has been recommended that MADs be used rather than no therapy in OSA patients, and the oral appliance should be customized to each patient [50].

Surgical Treatment

Uvulopalatopharyngoplasty is the process of widening the airway by removing part of the soft palate, uvula, and tonsils. In contrast, maxillomandibular advancement is the surgical process of reshifting the upper and lower jaws forward to enlarge the airway. The AASM guides how to approach surgical treatments in OSA patients. It recommends that clinicians evaluate patients for surgical treatment and assess factors that might impact the surgical outcome [40]. Overall, surgical treatment is considered to be an alternative for patients who cannot tolerate positive airway pressure. The Sleep Apnea Multilevel Surgery (SAMS) randomized clinical trial compared the effect of multilevel upper airway surgery on the AHI and Epworth Sleepiness Scale to the standard of care. Preliminary data demonstrated that in adults with moderate-to-severe OSA, combined palatal and tongue surgery compared to the standard treatment reduced the number of apnea and hypopnea events and patient-reported sleepiness at six months [51].

Hypoglossal Nerve Stimulation

Hypoglossal nerve stimulation can be described as a pacemaker-like device that provides neurostimulation to the branches of the hypoglossal nerve which is activated when breathing starts. The activation of the device leads to tongue protrusion, which opens the pharyngeal airway and prevents obstruction [52,53]. The Stimulation Therapy for Apnea Reduction (STAR) trial studied the effects of hypoglossal nerve stimulation treatment for moderate-to-severe patients. Primary outcomes included AHI, self-reported measures of sleepiness, sleep-related quality of sleep, snoring, and other polysomnography measures. At the 36-month follow-up, AHI events decreased from a median of 28.2 events per hour to 6.2 events per hour [54]. Although hypoglossal nerve stimulation appears to be beneficial, patient selection and screening should be conducted to identify ideal candidates. The best candidates are those with a BMI of 32, moderate-to-severe OSA, and absence of complete centric collapse at the soft palate level. Moreover, drug-induced sleep endoscopy should be done to identify the site of the collapse [55,56].

## Conclusions

OSA remains a prevalent chronic disease that continues to be underdiagnosed and untreated. OSA has multifactorial pathophysiology causing an increased risk of CVD states, metabolic dysfunction, mortality, and reduced quality of life due to a multitude of factors. The prevalence of OSA is expected to rise globally due to increasing obesity and the aging population in the next 30 years. Clinicians need to become familiar with the epidemiology and pathophysiology to efficiently screen for OSA and initiate effective treatment to prevent further health complications.
